# Optimizing surgical outcomes in bladder cancer patients undergoing radical cystectomy

**DOI:** 10.3389/fsurg.2022.1008318

**Published:** 2023-03-14

**Authors:** Maria Pere, Akara Amantakul, Supon Sriplakich, Tatum Tarin

**Affiliations:** ^1^ Department of Urology, University of Pittsburgh Medical Center, Pittsburgh, PA, United States; ^2^ Division of Urology, Chiang Mai University, Chiang Mai, Thailand

**Keywords:** bladder cancer (BC), radical cystectomy (RC), surgical outcomes, robotic vs. open cystectomy, lymph node dissection (LND)

## Abstract

**Purpose:**

To evaluate predictors of high-quality surgery and their effect on surgical outcomes in patients with bladder cancer undergoing radical cystectomy.

**Evidence acquisition:**

A systematic and thorough review was performed to identify the most recent literature on current optimal management and predictors of high-quality surgery for patients undergoing radical cystectomy.

**Conclusions:**

Muscle-invasive bladder cancer is an aggressive cancer requiring efficient and high-quality surgery in order to achieve the best oncological outcomes. Negative surgical margins, number of lymph nodes resected, lymph node dissection template, and surgical volume have been associated with improved oncologic outcomes. Robotic radical cystectomy continues to evolve and recent randomized controlled trials have shown that oncological outcomes are non-inferior when compared to the open technique. Regardless of approach, surgical technique should continually be evaluated and refined to optimize outcomes in patients undergoing radical cystectomy.

## Introduction

According to the SEER database, bladder cancer (BC) has the 6th highest incidence of all malignancies in the United States (US) with an estimated 81,180 new cases and 17,100 deaths in 2022 (1). BC is the tenth leading cause of death in the US. Of note, 20%–30% of newly diagnosed BC patients will present with muscle-invasive BC at the time of initial presentation. For these patients, radical cystectomy (RC) and bilateral pelvic lymph node dissection (LND) remains the gold standard of therapy. Surgical quality has a significant effect on peri-operative outcomes as well as cancer specific outcomes. Although there is no standard definition of a high-quality RC, other series have defined predictors of surgical quality using pathologic factors (negative margins, LND and number of nodes) (2–13), peri-operative management, and technical factors (surgical volume & open vs. robotic techniques). We will focus on surgical outcomes in patients with the above-mentioned pathology, specifically focusing on the predictors of high-quality surgery and their effects on surgical outcomes.

## Pathologic factors affecting surgical quality

### Surgical margins

Surgical margin status has long been established as an independent prognostic indicator and thus, a surrogate for surgical quality. In 2004, Herr et al. sought to evaluate whether surgical factors from patients enrolled in the SWOG 8,710 trial predicted BC outcomes. The endpoints of the study were post-cystectomy survival (PCS) and local recurrence (LR). They showed that negative margins were associated with longer PCS ([HR]: 0.37; *p* = 0.0007) and positive margin was an independent predictor of LR ([OR]: 11.2, *p* = 0.0001) (2). Another study completed out of Memorial Sloan Kettering Cancer Center retrospectively reviewed 1,589 patients who underwent RC and reported a positive soft tissue surgical margin (STSM) in only 4.2% of patients. It is important to note that in patients with organ confined disease, positive surgical margins were seen in 0% of patients. Importantly, they found that a positive surgical margin was an independent predictor of a higher rate of local disease relapse, distant metastases and lower disease specific survival (DSS), with DSS at five years being 32% in the positive STSM group and 72% in the negative STSM group (3). Additionally, in a large meta-analysis including 38,384 patients who had undergone RC, a positive surgical margin rate of 11% was reported as well as a significant association of positive margin rate with cancer-specific survival (CSS), recurrence free survival (RFS) and overall survival (OS) (4). Specifically, in this meta-analysis the summary relative risk estimate of positive surgical margins, and RFS, CSS and OS were 1.63 (95% CI, 1.46–1.83), 1.82 (95% CI, 1.63–2.04) and 1.68 (95% CI, 1.58–1.80), respectively compared with negative surgical margins. This meta-analysis had many strengths. First, it included 36 studies with large sample sizes. Secondly, strict accordance with their inclusion and exclusion criteria was maintained. Several limitations with this study are also noted with the most important being most of the included studies were retrospective, rendering their meta-analysis sensitive to potential confounding variables. In 2019 Rai, et al. compared robotic vs. open RC for BC in adults and found that positive surgical margins, as a surrogate for oncological outcome are comparable between robotic and open RC, although with low certainty (5). The above data along with numerous other retrospective studies reiterate the importance of negative surgical margins for better oncologic outcomes in these patients. After diagnosis, only treatment related parameters can alter the likelihood of recurrence, therefore we stress the importance of wide peri-vesical dissection to avoid a positive margin in RC patients.

### Number of lymph nodes (LNs)

The number of LNs needed for a dissection to be considered adequate has not been clearly established in the literature. Herr et al. in 2004 found that in patients in whom at least ten LNs were removed, five-year survival improved from 44% to 61% (2). Furthermore, in a study that included 1,121 patients who underwent RC in a 14-year period, Koppie et al. showed that a higher number of dissected LNs correlated with improved survival rates (6). Additionally, in 2008, Wright et al. found that removal of >10 LNs was associated with increased OS (7). It should be noted that all of the current studies that try to establish a minimum number of LNs needed for an adequate dissection are all retrospective or non-randomized reports so further studies are needed to help establish minimal LNs. Currently, the AUA/ASCO/ASTRO/SUO (14) guidelines state that a minimum of 10 LNs should be attained at the time of LND (14).

### Extent of bilateral pelvic lymph node dissection

A meticulous LND at the time of RC is paramount to achieve the best oncological outcome. Approximately 25% of patients will have pathologic LN metastases at the time of RC (8) and LN status has been shown to be a powerful surrogate for long-term recurrence free and OS following RC (9). Herr et al. in 2004 showed that the extent of LND is an independent predictor of survival and local recurrence (2). It is clear that a LND should be performed at the time of RC with a therapeutic role, +/− diagnostic/staging role. Importantly, the anatomic template that should be performed remains controversial. Currently, a “standard template” dissection, according to the European Association of Urology (EAU) (15)/National Comprehensive Cancer Network (NCCN) (16) guidelines entails removal of all lymphatic tissue around the common iliac, internal and external iliac, and obturator packets. In cases of advanced disease, an extended dissection can be performed, and multiple studies have demonstrated that this offers improved prognostic staging (10–12). In 2004, Bochner et al. reported on the anatomic extent and spread of LN metastases. They conducted a prospective evaluation on 144 patients who underwent either standard LND (pelvic) or extended LND between June 2001 and April 2003. The absolute number of positive LNs was significantly higher in the extended group (22.5 vs. 8), however both groups yielded the same percentage of patients with positive nodes (21%) demonstrating no staging advantage. A subset analysis of patients with unexpected microscopic nodal involvement revealed that 33% had involvement of the common iliac nodes, leading the authors to conclude that this area should be considered part of a standard LND (10). Furthermore, in a study of 591 patients over a ten-year period, the same group reported LN involvement in 19% of patients. In this series, seven patients (6%) demonstrated skip lesions with positive nodes above the common iliac bifurcation, with no positive LNs within the true pelvis. They concluded this further adds to the data that common iliac LNs should be included as part of a standard LND (11). In 2008, Dhar et al. published a retrospective study comparing limited LND to extended LND across two centers. In this study, 26% node positive patients were identified in the extended template and 13% node positive patients were found in the limited template. For node positive patients, the 5-year RFS was 7% for limited template and 35% for extended LND. This large cohort study confirmed the value of extended LND in regard to staging accuracy and prognosis (12).

The first prospective randomized control trial (RCT) comparing standard LND vs. extended was completed in 2018 by Gschwend et al. and is known as the LEA trial. They defined limited dissection as obturator, internal and external iliac nodes with extended dissection including limited + deep obturator, presacral, para-caval, inter-aorto-caval and para-aortal nodes. Their results (extended LND *n* = 198, standard LND *n* = 203) did not demonstrate a 5-year difference in recurrence-free or cancer specific survival between these two groups, however the extended arm did demonstrate non-significant absolute improvement in these categories (69.3% vs. 62% and 77.5% vs. 66.2%, respectively) (13). A confounding factor in the study was that none of these patients received neoadjuvant chemotherapy. Additionally, the high percentage of pT1 disease (14%) could have limited the results significance since more extensive LND typically benefits those with more advanced disease. Lastly, a limitation of this study was that it was not powered to demonstrate the non-inferiority of standard vs. extended LND. Nonetheless, this study represents the best level of evidence currently available, therefore we conclude with the current literature that at minimum a standard LND must be performed. It is important to note that a “standard” LND is defined differently by different guidelines. The EAU (15) and NCCN (16) guidelines include the common iliac LNs whereas the AUA/ASCO/ASTRO/SUO (14) guidelines do not mandate the common iliac LNs be included. The results regarding the utility of extended LND are anticipated from the SWOG 1,011 trial with results expected in 2022.

## Peri-Operative management and effect on surgical outcomes

Surgical quality is not only defined as operative technique but must include peri-operative management and length of hospital stay as well. Enhanced recovery after surgery (ERAS) protocols have been developed to standardize perioperative management of patients undergoing RC and shortening length of hospital stay while minimizing complications.

### Enhanced recovery after surgery (ERAS) protocols and shorter length of stay (LOS)

ERAS protocols were first introduced in colorectal surgery, however, more recently, these ERAS protocols have become crucial in urologic-oncology, more specifically, in the RC population. These protocols include avoidance of mechanical bowel preparation, judicious intra-operative fluid use, use of Alvimopan to help reduce post-operative ileus, early advancement of diet, VTE prophylaxis, early mobilization and avoidance of narcotics. These measures have been shown to shorten hospital stay and improve outcomes (17, 18). A study in 2008, completed by Arumainayagam et al., studied this protocol specifically in RC patients. They evaluated 56 non-ERAS patients to 56 patients who did follow the ERAS protocol. The LOS was reported to be shorter in the ERAS group (13 days vs. 17 days). However, the readmission, morbidity and mortality rates were not significantly different between the groups (19). More recently, a meta-analysis completed in 2020 by Williams et al. showed that ERAS use was associated with reduced morbidity, quicker bowel recovery, and shorter LOS, without affecting mortality. This group also found individual ERAS components associated with shorter LOS included avoiding nasogastric tube use and utilization of local anesthetic blocks (20). Further, a phase IV study by Kauf et al. in 2014 looking specifically at the use of the *μ*-opioid receptor, Alvimopan, in cystectomy patients demonstrated a 2.63 day reduction in hospital stays, lower rates of TPN for post-operative ileus (10% vs. 25%), as well as a significant cost savings per patient (18). Standardization of perioperative care with ERAS for patients undergoing RC has decreased morbidity and LOS without increasing complications or affecting mortality.

## Technical factors affecting surgical quality

Many studies have shown that institutions with higher volume of complex surgeries will have better surgical outcomes. While RC has traditionally been approached *via* an open technique, developments in minimally invasive surgery have continued to progress and have shown potential advantages in the areas of blood loss and complications, thus, the transition to robotic techniques has been evolving.

### Surgical volume

A meta-analysis completed by Goossens-Laan et al. in 2011 concluded that post-operative mortality after RC is significantly inversely associated with high-volume providers (21). In this meta-analysis, two studies concerning surgical volume and post-operative mortality showed a significant effect in favor of high-volume surgeons (OR: 0.55, 95% CI, 0.41–0.73 and OR: 0.64; 95% CI, 0.44–0.91, respectively) (21–23). Furthermore, Afshar et al. in 2018 evaluated all RCs in England from 2003 to 2014 after centralization was encouraged following implementation of the “Improving Outcomes Guidelines” (IOG). A key recommendation of these guidelines was to centralize RCs to high output centers which they defined as institutions/teams which serve populations of one million or more and additionally carry a cumulative total of ≥50 operations/year. In their study, they found that procedures adhering to the IOG had better 30-day mortality (2.1% vs. 2.9%; *p* = 0.003) than those that did not, and better 1-year mortality (21.5% vs. 25.6%; *p* < 0.001), LoS (14d vs. 16d; *p* < 0.001) and re-intervention rates (30.0% vs. 33.6%; *p* < 0.001) (24). These above data suggest that patients have better surgical and oncological outcomes when served by high-volume centers adding to the growing consensus that these complex surgeries should be performed at these institutions.

### Open radical cystectomy (ORC) vs. Laparoscopic (LRC) and robotic radical cystectomy (RARC)

The first minimally invasive RC was published as LRC in 1993 by Badajoz et al (25). After that, several studies were published and showed that LRC is technically achievable and safe (26–28). This technique presented many perioperative advantages including lower estimated blood loss, lower need for transfusion, less opioid requirements, fewer post-operative complications and shorter postoperative convalescence period (29–31). Most importantly, the long-term oncologic outcome was comparable to open surgery in many studies (32–34). The most recent study was published in 2021 by Huang et al. and was a single-institution retrospective trial comparing laparoscopic and open RC and LND with a primary endpoint of survival outcomes in 607 patients. The results showed that the LRC group had less estimated blood loss (*p* < 0.001 and *p* < 0.001) and fewer complications (*p* < 0.001 and *p* = 0.008). There was no difference in the overall survival (*p* = 0.216 and *p* = 0.961) and progression-free survival (*p* = 0.826 and *p* = 0.462) (35). However, most of the evidence for LRC is limited to the retrospective series. Nonetheless, this technique can be applied to institutions and health systems that cannot afford the high cost of robotic surgery.

Many retrospective studies have attempted to compare open vs. robotic RC. However, the first randomized control trial in this area was completed by Bochner. This was a single-institution randomized trial comparing robotic and open RC and LND with primary endpoint of 90-day complications. Between 2010 and 2013, patients were randomized to ORC or RARC with LND with both groups undergoing extracorporeal diversions. The study closed at the mandated interim analysis because of futility after demonstrating a similar grade complication rate (62% robotic, 66% open). The by-randomization and the intention-to-treat-analysis demonstrated similar pathological outcomes and the RFS and CSS were similar between the robot and open surgery arms (*p* = 0.4). Risk of recurrence at 5 yr was 36% and 41% for RARC and ORC respectively (difference: −5.2%; 95% CI: 125–14). The wide CIs around the difference in recurrence risks precluded them from making conclusions regarding oncological equivalence of the surgical modalities, highlighting a major limitation of this trial, which is that it was not powered to determine differences in cancer recurrences, survival outcomes or patterns of recurrence. Similarly, the difference in rate of abdominal recurrence did not meet conventional levels of significance (sHR: 0.38; 95% CI: 0.07–1.96; *p* = 0.2). However, when the pelvic and abdominal recurrences were combined into a single group representing local/regional recurrence, the ORC group showed significantly less local/regional recurrence compared to RARC (sHR: 0.34; 95% CI: 0.12–0.93; *p* = 0.035). When looking more closely at these patterns, three RARC patients with local recurrences had soft tissue disease with direct rectosigmoid invasion. This pattern of pelvic recurrence was not identified in the ORC arm. All 5 RARC patients who relapsed within the abdomen demonstrated invasion of the abdominal wall with synchronous bowel implants, another pattern not identified in those undergoing ORC raising concerns for cancer recurrence due to laparoscopic technique (36).

The strongest level of evidence supporting the oncological efficacy of RARC is the RAZOR study. This study is a randomized, open-label, non-inferiority phase 3 trial done at 15 medical centers across the US. The trials primary end-point was progression free survival at 2 years after surgery. Patients were eligible if they were aged 18 years or older and had biopsy proven clinical stage T1-T4, N0-N1, M0 bladder cancer or refractory carcinoma *in situ*. Patient who had previously had open abdominal or pelvic surgery or who had any pre-existing health conditions that would preclude safe initiation or maintenance of pneumoperitoneum were excluded. Ultimately, 302 patients (159 patients in the robotic cystectomy group and 153 patients in the open cystectomy group) were included in the modified intention-to-treat analysis set. Two-year progression free survival was 72.3% (95% CI 64.3 to 78.8) in the RARC group and 71.6% (95% CI 63.6 to 78.2) in the ORC group (difference 0.7% (95% CI –9.6 to 10.9, showing a non-inferiority = 0.001); showing non-inferiority of RARC to ORC (37). The results of the sensitivity analysis in the modified intention to treat population also confirmed the non-inferiority of robotic cystectomy. EBL and median length of hospital stay were significantly lower in the robotic group. Of note, median operating time was significantly longer in the RARC group. No significant differences in overall complications were identified between treatment groups. No significant differences were identified between the treatment groups in tumor histology and staging, extended LND, the mean number of lymph nodes removed, and proportion of patients with positive surgical margins. Of the patients with positive surgical margins, 78% in the RARC group and 71% in the ORC group had stage T3 BC or higher (37).

In 2020 this same group published an update at 3 years, with the study endpoints of time to recurrence, progression free survival and overall survival. At 36 months progression free survival (PFS) and OS were comparable in the 2 groups (*p* = 0.756 and *p* = 0.432, respectively). The estimated progression-free rate at 36 months was 68.4% in the robotic group and 65.4% in the open group. In conclusion, at three years this analysis from the updated RAZOR trial shows no difference in the cumulative incidence of recurrence, PFS or OS for RARC vs. ORC (38). In 2022, Catto et al. published a RCT evaluating 338 patients undergoing RARC with intracorporeal diversion vs. ORC with the objective of comparing recovery and morbidity. The primary outcome was the number of days alive and out of the hospital within 90 days of surgery. The median number of days alive and out of the hospital within 90 days of surgery was 82 for RARC vs. 80 for ORC [adjusted difference, 2.2 days (95% CI, 0.50–3.85); *p* = 0.01] (39). The median length of stay in the hospital in the hospital was 7 days for RARC and 8 days for ORC; adjusted difference, 1.11 days ([95% CI, 0.002–2.22 days]; *p* = 0.05). The authors concluded that among patients with non-metastatic BC undergoing RC, treatment with RARC with intracorporeal diversion vs. ORC resulted in a statistically significant increase in days alive and out of the hospital over 90 days (39).

## Conclusions

Muscle-invasive BC is an aggressive cancer with poor outcomes if not treated efficiently and with high quality surgery. Negative surgical margins, LND including at least 10 lymph nodes as well as a standard-LND (common iliac, internal, and external iliac and obturator packets bilaterally per EAU (15)/NCCN (16) guidelines) are required for optimal outcomes. The importance of the extended LND remains controversial, and the results of the SWOG 1,011 trial are pending and could help further solidify the role of extent of LND. RC is a complex operation best managed by high-volume surgeons at high volume centers. The technique for RC continues to evolve over time, and thus far, randomized trials for RARC vs. ORC have shown that robotic oncologic outcomes are non-inferior to open cystectomies, however, further data is needed. Nonetheless, surgical quality can have significant impact on perioperative and oncologic outcomes, and therefore high-quality surgery must be emphasized regardless of surgical technique.
